# Prognostic Value of Pretreatment Overweight/Obesity and Adipose Tissue Distribution in Resectable Gastric Cancer: A Retrospective Cohort Study

**DOI:** 10.3389/fonc.2021.680190

**Published:** 2021-06-24

**Authors:** Lihu Gu, Yangfan Zhang, Jiaze Hong, Binbin Xu, Liuqiong Yang, Kun Yan, Jingfeng Zhang, Ping Chen, Jianjun Zheng, Jie Lin

**Affiliations:** ^1^ Department of General Surgery, HwaMei Hospital, University of Chinese Academy of Sciences, Ningbo, China; ^2^ Ningbo Institute of Life and Health Industry, University of Chinese Academy of Sciences, Ningbo, China; ^3^ Key Laboratory of Diagnosis and Treatment of Digestive System Tumors of Zhejiang Province, HwaMei Hospital, University of Chinese Academy of Sciences, Ningbo, China; ^4^ Ningbo University School of Medicine, Ningbo, China; ^5^ The Second Clinical Medical College, Zhejiang Chinese Medical University, Hangzhou, China; ^6^ Department of Nutrition, HwaMei Hospital, University of Chinese Academy of Sciences, Ningbo, China; ^7^ Department of Radiology, HwaMei Hospital, University of Chinese Academy of Sciences, Ningbo, China; ^8^ Department of Thoracic Surgery, HwaMei Hospital, University of Chinese Academy of Sciences, Ningbo, China

**Keywords:** overweight, obesity, adipose tissue distribution, gastric cancer, prognosis

## Abstract

**Background:**

This is a study aimed at exploring the relationship between pretreatment overweight/obesity, adipose tissue distribution, and long-term prognosis of gastric cancer.

**Methods:**

A total of 607 gastric cancer patients were involved in the retrospective cohort study. Overweight/obese patients were defined as body mass index (BMI) greater than 25 kg/m^2^, and adipose tissue distribution parameters, including visceral adipose tissue (VAT), subcutaneous adipose tissue (SAT), and VAT/SAT ratio were measured at the level of the third lumbar vertebra using computerized tomography images within 15 days before the surgery. Multiple Cox regression models were applied to evaluate the association between overweight/obesity and disease-specific survival (DSS) of gastric cancer, and covariates including age, gender, T stage, N stage, and chemotherapy were adjusted. Furthermore, multiple Cox regression models were performed to evaluate the association between adipose tissue distribution parameters and DSS of gastric cancer; except for covariates mentioned above, overweight/obesity was adjusted additionally.

**Results:**

Overweight/obesity was a predictive factor (HR = 0.61, 95% CI: 0.37–0.99) for the prognosis of gastric cancer. After additionally adjusting for overweight/obesity, high SAT percentage was an independent protective factor (HR = 0.59, 95% CI: 0.36–0.96), while high VAT percentage (HR = 1.68, 95% CI: 1.06–2.68) and high VAT/SAT ratio (HR = 1.99, 95% CI: 1.19–3.34) were independent risk factors for DSS of gastric cancer. Compared with other patients (overweight/obesity with low VAT/SAT ratio group, non-overweight/obesity or high VAT/SAT ratio group), patients in the non-overweight/obesity with high VAT/SAT ratio group had a worse prognosis (HR = 1.89, 95% CI: 1.28–2.77).

**Conclusion:**

These results suggest that overweight/obesity is a predictive factor for the prognosis of gastric cancer. The VAT/SAT ratio could be used as a promising prognostic factor for gastric cancer. Therefore, in preoperative evaluation of gastric cancer patients, attention should be paid not only to BMI but also to adipose tissue distribution.

## Introduction

Gastric cancer is the fifth most commonly diagnosed malignancy and the fourth leading cause of death from cancer worldwide (1). At present, the main treatment for resectable gastric cancer is surgery combined with adjuvant therapy, especially chemotherapy (2). However, the long-term prognosis of advanced gastric cancer is not satisfactory (3). The correlation between obesity and the prognosis of gastric cancer has always been controversial (4). Previous studies have shown that obese patients with gastric cancer have better long-term survival than non-overweight/obese patients (5, 6). However, several studies have shown that obesity is not associated with survival in gastric cancer (7–9). In most studies, obesity was used as an adjuvant parameter and was not further analyzed. Besides, when considering the association between obesity and the prognosis of gastric cancer patients, we should not only consider “obesity” defined by body mass index (BMI), but also consider the relationship between the adipose tissue distribution and gastric cancer, including the relationship between the content or distribution of adipose tissue and gastric cancer.

There are certain differences in the adipose tissue distribution; even in the same BMI population, the adipose tissue distribution in the body is not the same. However, studies on the effect of adipose tissue distribution on the long-term prognosis of gastric cancer are largely lacking. Human adipose tissue mainly includes visceral adipose tissue (VAT) and subcutaneous adipose tissue (SAT), and their effects on tumors vary (10). Although obesity was initially found to confer a survival advantage in cancer patients, mounting evidence suggests that increased visceral adipose tissue may negatively influence survival in patients with numerous cancers (11, 12). In contrast, current studies have found that SAT is protective and associated with the prognosis of various tumors, including colorectal cancer (13), prostate cancer (14), head and neck cancer (15), and hepatocellular carcinoma (16).

In recent years, increased visceral adiposity has been associated with the prognosis of various tumors (17–19). However, the association between visceral adiposity and the prognosis of gastric cancer involves retrospective studies with small sample sizes, and the results remain controversial (20). Computed tomography (CT) scan is routinely used in the diagnosis and treatment of gastric cancer. Besides, CT is the gold standard method for adipose tissue component analysis due to its accuracy (21). However, few studies have used CT to evaluate the adipose tissue distribution and its correlation with prognosis in gastric cancer patients. Therefore, this study used CT to evaluate the preoperative adipose tissue distribution of patients with resectable gastric cancer and explored the relationship between adipose tissue distribution and long-term prognosis in gastric cancer.

## Methods

### Participants

A total of 607 patients who underwent radical gastrectomy from January 2013 to December 2017 at HwaMei Hospital, University of Chinese Academy of Sciences, were included in this retrospective observational cohort study. The inclusion criteria were as follows: (1) histologically proven primary adenocarcinoma of the stomach; (2) no previous history of gastrectomy or other malignant tumors; (3) pathologically negative resection margins (R0 resection) and lymphadenectomy (D1 or more). Exclusion criteria include the following: (1) The patient had received neoadjuvant chemotherapy or chemo-radiotherapy before surgery; (2) The patients had postoperative survival time less than 30 days; (3) Patients were followed up for less than 36 months. This study was approved by the Ethics Committee of the HwaMei Hospital, University of Chinese Academy of Sciences (approval NO. PJ-NBEY-KY-2019-153-01). Written informed consent was obtained from all the participants.

### Histological Examination

The surgical specimens were assessed according to the handling guideline of the 3rd edition of the Japanese classification of gastric carcinoma (22) and confirmed by three senior pathologists, specialists in gastric cancer. Routine pathology in the department of pathology included the use of *pro forma* reports and whole-mount slides. Information on pathological variables, including tumor location, differentiation, perineural invasion, lymph vascular invasion, and tumor size, were obtained from the histopathological reports. T and N stages were classified according to the 8th edition of the (Union for International Cancer Control/American Joint Committee on Cancer) UICC/AJCC TNM staging system (23).

### Demographic and Clinical Parameters

Demographic and clinical characteristics, including age, gender, gastrectomy, and postoperative chemotherapy, were retrieved within 24 h after hospitalization. Weight and height were measured with participants without shoes and were recorded to the nearest 0.1 kg and 0.1 cm, respectively. BMI was calculated as body weight/height squared (kg/m^2^). In general, Asian populations have a smaller physique than Western individuals and tend to suffer from metabolic complications of obesity at a lower BMI than others, thus overweight/obese patients were defined as BMI ≥25 kg/m^2^ in the present study (24).

### Measurement of Adipose Tissue Distribution Parameters

**Figure 1:**
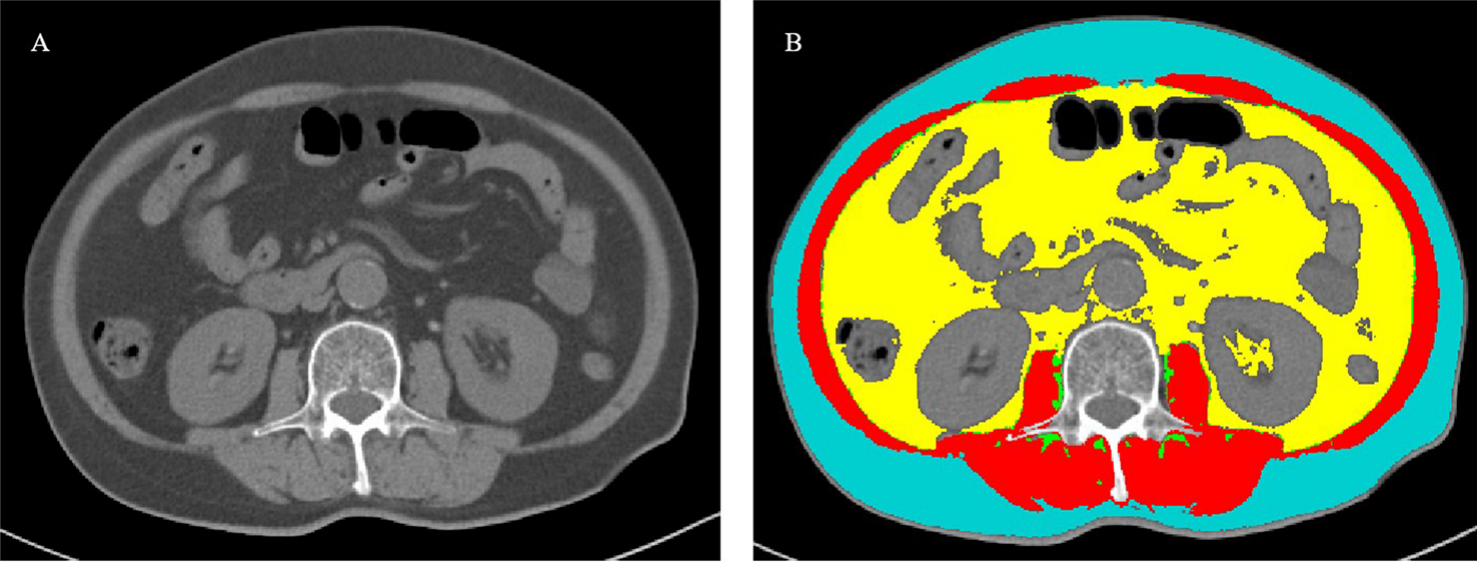
Measurement of body composition parameters with cross-sectional CT images at the third lumbar level. **(A)** A cross-sectional CT image at the third lumbar level. **(B)** Body composition automatically calculated using the ABACS Auto Segmentation module in SliceOmatic software (ver. 5.0). Visceral adipose tissue, subcutaneous adipose tissue, intramuscular adipose tissue, and muscle were targeted as yellow, blue, green, and red, respectively.

CT images were taken within 15 days before surgical resection and then analyzed. The level of the third lumbar vertebra landmark was independently identified by two experienced radiologists. The corresponding single axial image was extracted and saved as a DICOM image file. ABACS Auto Segmentation module in SliceOmatic software (ver. 5.0) was used to measure the patient’s adipose tissue distribution parameters, including the cross-sectional area of VAT, SAT, and intramuscular adipose tissue (**Figure 1**). The total fat area was equal to the sum of VAT, SAT, and intramuscular adipose tissue areas. Percentages of VAT and SAT were calculated by dividing VAT area and SAT area divided by total fat area (cm^2^/cm^2^), respectively. The VAT/SAT ratio was calculated by dividing the VAT area by SAT area.

### Follow-Up

All patients were followed up every 3–6 months for the first 2 years and annually thereafter until death or at least 5 years after the surgery. Disease-specific survival (DSS) was defined as the time from surgery to death of gastric cancer. Cases in which patients were lost during follow-up or died of other diseases were regarded as censored, and the date of their last known contact was recorded. The median follow-up period for the present cohort was 50 months (range 3–95 months) and the follow-up was closed in December 2020.

### Statistical Analysis

The characteristics of the patients are presented as median (interquartile range) for continuous variables and frequency (percentages) for categorical variables. The differences between overweight/obese and non-overweight/obese patients were tested using the Wilcoxon rank-sum test for continuous variables and chi-squared tests or Fisher’s exact test for categorical variables. The optimal cutoff points for VAT and SAT percentages and VAT/SAT ratio were determined using maximally selected log-rank statistics [R packages (maxstat)]. The DSS rate was analyzed using the Kaplan–Meier method and the log-rank test was used to determine significant differences between groups. The association between overweight/obesity and DSS was examined using multiple Cox regression models after adjusting for age, gender, T stage, N stage, and chemotherapy. The relationships between adipose tissue distribution parameters, and DSS were analyzed after additionally adjusting for overweight/obesity. We further divided patients into three subgroups based on overweight/obesity and VAT/SAT ratio categories: 1) Overweight/obese patients with low VAT/SAT ratio, 2) non-overweight/obese patient with low VAT/SAT ratio or overweight/obese patients with high VAT/SAT ratio, and 3) non-overweight/obese patient with high VAT/SAT ratio. The differences among these groups were tested using analyses of variance for continuous variables and chi-squared tests or Fisher’s exact test for categorical variables. The multiple Cox regression model was reconducted to analyze the association between overweight/obesity and VAT/SAT ratio categories and DSS. All p-values were two-tailed, and statistical significance was defined as p <0.05. Statistical analyses were performed using STATA 15.1.

## Results

### Patient Characteristics Based on Overweight/Obesity

The baseline characteristics and classification of patients in the entire cohort are summarized in **Table 1**. Among the 607 patients, the number of male patients was more than twice that of female patients. The tumors in the distal stomach accounted for 80% of all the tumors. Patients with gastric cancer stages I, II, and III were 197, 112, and 298, respectively. The 5-year disease-specific survival rates of patients in the present study was 79.9%, and were 97.6, 86.1, and 68.0% for patients in stages I, II, and III, respectively (data not shown).

**Table 1:** Characteristics of entire cohort and classified by overweight/obesity.

	Total	Overweight/obesity	Non-overweight/obesity (N = 493)	P value
	(N = 607)	(N = 114)		
Age (>65 years)	268 (44.15)	46 (40.35)	222 (45.03)	0.365
Male	428 (70.51)	77 (67.54)	351 (71.20)	0.441
BMI	22.1 (19.79, 24.2)	26.79 (25.71, 27.7)	21.28 (19.20, 23.03)	**<0.001**
Tumor location				0.058
Upper third	60 (9.88)	18 (15.79)	42 (8.52)	
Middle third	78 (12.85)	12 (10.53)	66 (13.39)	
Lower third	461 (75.95)	84 (73.68)	377 (76.47)	
Two-thirds or more	8 (1.32)	0 (0.00)	8 (1.62)	
Gastrectomy				0.541
Distal	488 (80.40)	88 (77.19)	400 (81.14)	
Total	118 (19.44)	26 (22.81)	92 (18.66)	
Proximal	1 (0.16)	0 (0.00)	1 (0.20)	
Tumor size (>5 cm)	148 (24.38)	28 (24.56)	120 (24.34)	0.961
Differentiated	313 (51.57)	66 (57.89)	247 (50.10)	0.133
Perineural invasion	239 (39.37)	47 (41.23)	192 (38.95)	0.653
Lymph vascular invasion	283 (46.62)	55 (48.25)	228 (46.25)	0.700
T category				**0.022**
T1	171 (28.17)	31 (27.19)	140 (28.40)	
T2	72 (11.86)	12 (10.53)	60 (12.17)	
T3	12 (1.98)	6 (5.26)	6 (1.22)	
T4a	339 (55.85)	60 (52.63)	279 (56.59)	
T4b	12 (2.14)	5 (4.39)	8 (1.62)	
N category				0.960
N0	255 (42.01)	48 (42.11)	207 (41.99)	
N1	94 (15.49)	18 (15.79)	76 (15.42)	
N2	120 (19.77)	20 (17.54)	100 (20.28)	
N3a	106 (17.46)	22 (19.30)	84 (17.04)	
N3b	32 (5.27)	6 (5.26)	26 (5.27)	
Chemotherapy	401 (66.06)	82 (71.93)	319 (64.71)	0.142
Adipose tissue distribution parameters				
SAT area	91.72 (56.35,126.60)	130.85 (100.70,163.00)	81.89 (49.92, 114.30)	**<0.001**
VAT area	94.17 (45.00,149.40)	171.55 (114.90, 222.70)	80.88 (36.17,130.20)	**<0.001**
Total fat area	205.47 (118.86,292.48)	321.52 (261.11,286.37)	183.53 (99.90, 252.02)	**<0.001**
SAT percent	0.47 (0.38, 0.58)	0.43 (0.34, 0.55)	0.48 (0.39, 0.60)	**0.002**
High SAT percent	88 (14.50)	14 (12.28)	74 (15.01)	0.456
VAT percent	0.47 (0.35, 0.58)	0.55 (0.43, 0.63)	0.46 (0.33, 0.56)	**<0.001**
High VAT percent	505 (83.20)	106 (92.98)	399 (80.93)	**0.002**
VAT/SAT ratio	1.01 (0.60, 1.50)	1.27 (0.79, 1.83)	0.96 (0.56, 1.44)	**<0.001**
High VAT/SAT ratio	529 (87.15)	108 (94.74)	421 (85.40)	**0.007**

BMI, body mass index; SAT, subcutaneous adipose tissue; VAT, visceral adipose tissue.

P value for difference between groups in percentages (chi-square test or Fisher’s exact test) or rank sum (Kruskal–Wallis test).

Bold values represent statistically significant differences (P < 0.05).

There were no significant differences in demographic and clinical parameters except for T staging distribution between overweight/obesity groups. Compared with non-overweight/obese patients, overweight/obese patients had a low SAT percentage (p = 0.002), high VAT percentage (p < 0.001), and high VAT/SAT ratio (p < 0.001).

### Overweight/Obesity and DSS

The optimal cutoff values of SAT percentage, VAT percentage, and VAT/SAT ratio were 64, 30, and 44%, respectively (**Figure S1**).

**Figure 2:**
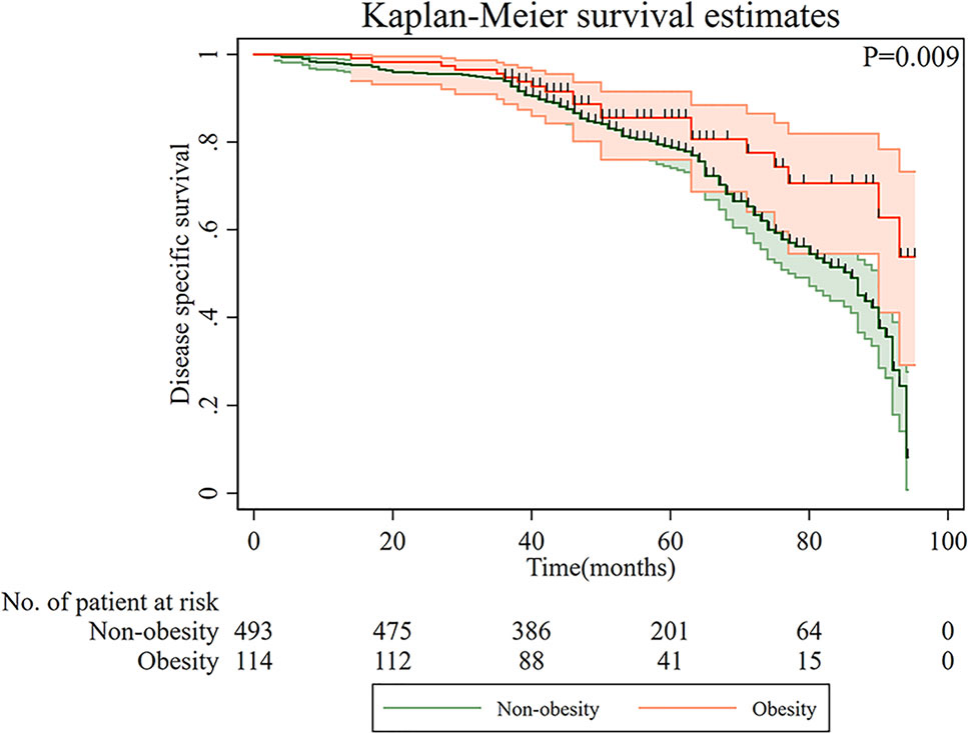
Disease-specific survival of patients with gastric cancer according to overweight/obesity.

Kaplan–Meier curves of DSS based on overweight/obesity are shown in **Figure 2**. Compared with non-overweight/obese patients, overweight/obese patients had a significantly better prognosis (p = 0.009). Furthermore, a multiple Cox regression model was conducted to examine the relationship between overweight/obesity and DSS (**Table 2**). After adjusting for age, gender, T categories, N categories, and chemotherapy, overweight/obesity was an independent protective factor for long-term DSS in gastric cancer patients (HR = 0.61, 95% CI: 0.37–0.99).

**Table 2:** Association between overweight/obesity and disease-specific survival by multivariate Cox analysis in patients with gastric cancer.

	HR (95%CI)	P value
Overweight/obesity	0.61 (0.37, 0.99)	**0.044**
Age (>65 years)	1.59 (1.14, 2.2)	**0.006**
Female	0.89 (0.63, 1.26)	0.506
T category		
T1		
T2	2.51 (0.88, 7.15)	0.084
T3	2.97 (0.57, 15.55)	0.197
T4a	3.81 (1.54, 9.42)	**0.004**
T4b	10.76 (3.47, 33.33)	**<0.001**
N category		
N0		
N1	2.34 (1.19, 4.63)	**0.014**
N2	2.33 (1.24, 4.38)	**0.009**
N3a	6.24 (3.37, 11.55)	**<0.001**
N3b	8.28 (4.12, 16.62)	**<0.001**
Chemotherapy	0.80 (0.53, 1.21)	0.294

BMI, body mass index; HR, hazard ratios; CI, confidence interval.

Bold values represent statistically significant differences (P < 0.05).

### Adipose Tissue Distribution and DSS

**Figure 3:**
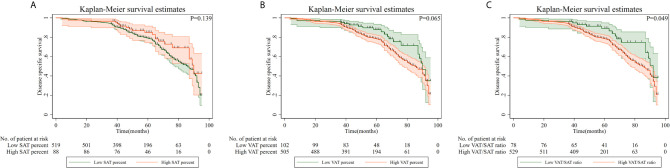
Disease-specific survival of patients with gastric cancer according to adipose tissue distribution parameters. **(A)** High SAT percentage; **(B)** High VAT percentage; **(C)** High SAT/VAT ratio. VAT, visceral adipose tissue; SAT, subcutaneous adipose tissue.

Kaplan–Meier curves of DSS based on adipose tissue distribution, including SAT and VAT percentages and VAT/SAT ratio are shown in **Figure 3**. Compared with patients with a low VAT/SAT ratio, patients with a high VAT/SAT ratio had a significantly worse prognosis (p = 0.049). Multiple Cox regression models were conducted to evaluate the relationship between adipose tissue distribution parameters and DSS (**Table 3**). After additionally adjusting for overweight/obesity, high SAT percentage was an independent protective factor (HR = 0.59, 95% CI: 0.36–0.96), while high VAT percentage (HR = 1.68, 95% CI: 1.06–2.68) and high VAT/SAT ratio (HR = 1.99, 95% CI: 1.19–3.34) were independent risk factors for DSS of gastric cancer.

**Table 3:** Associations between adipose tissue distribution parameters and disease-specific survival by multivariate Cox analysis in patients with gastric cancer.

	High SAT percent		High VAT percent		VAT/SAT ratio	
	HR (95%CI)	P value	HR (95%CI)	P value	HR (95%CI)	P value
Adipose tissue distribution parameter	0.59 (0.36, 0.96)	**0.032**	1.68 (1.06, 2.68)	**0.028**	1.99 (1.19, 3.34)	**0.009**
Overweight/obesity	0.56 (0.34, 0.91)	**0.021**	0.55 (0.34, 0.91)	**0.019**	0.55 (0.33, 0.90)	**0.017**
Age (>65 years)	1.56 (1.13, 2.17)	**0.008**	1.54 (1.11, 2.14)	**0.010**	1.56 (1.12, 2.16)	**0.008**
Female	0.98 (0.69, 1.4)	0.929	0.93 (0.66, 1.32)	0.696	0.95 (0.67, 1.35)	0.794
T category						
T1	1		1		1	
T2	2.51 (0.88, 7.13)	0.085	2.49 (0.87, 7.08)	0.880	2.49 (0.88, 7.07)	0.087
T3	2.77 (0.52, 14.66)	0.230	2.86 (0.54, 15.09)	0.216	2.81 (0.53, 14.77)	0.223
T4a	3.59 (1.45, 8.88)	**0.006**	3.61 (1.46, 8.95)	**0.006**	3.63 (1.47, 8.95)	**0.005**
T4b	10.44 (3.35, 32.47)	**<0.001**	10.39 (3.34, 32.31)	**<0.001**	10.73 (3.47, 33.20)	**<0.001**
N category						
N0	1		1		1	
N1	2.38 (1.21, 4.68)	**0.012**	2.31 (1.17, 4.57)	**0.016**	2.33 (1.18, 4.60)	**0.015**
N2	2.45 (1.3, 4.61)	**0.005**	2.35 (1.25, 4.43)	**0.008**	2.42 (1.29, 4.54)	**0.006**
N3a	6.74 (3.63, 12.51)	**<0.001**	6.55 (3.53, 12.14)	**<0.001**	6.65 (3.60, 12.30)	**<0.001**
N3b	9.01 (4.47, 18.15)	**<0.001**	8.65 (4.3, 17.4)	**<0.001**	8.96 (4.46, 18.01)	**<0.001**
Chemotherapy	0.82 (0.54, 1.25)	0.356	0.81 (0.54, 1.23)	0.329	0.82 (0.54, 1.24)	0.340

BMI, body mass index; SAT, subcutaneous adipose tissue; VAT, visceral adipose tissue; HR, hazard ratios; CI, confidence interval.

Bold values represent statistically significant differences (P < 0.05).

### Overweight/Obesity and VAT/SAT Categories and DSS

**Figure 4:**
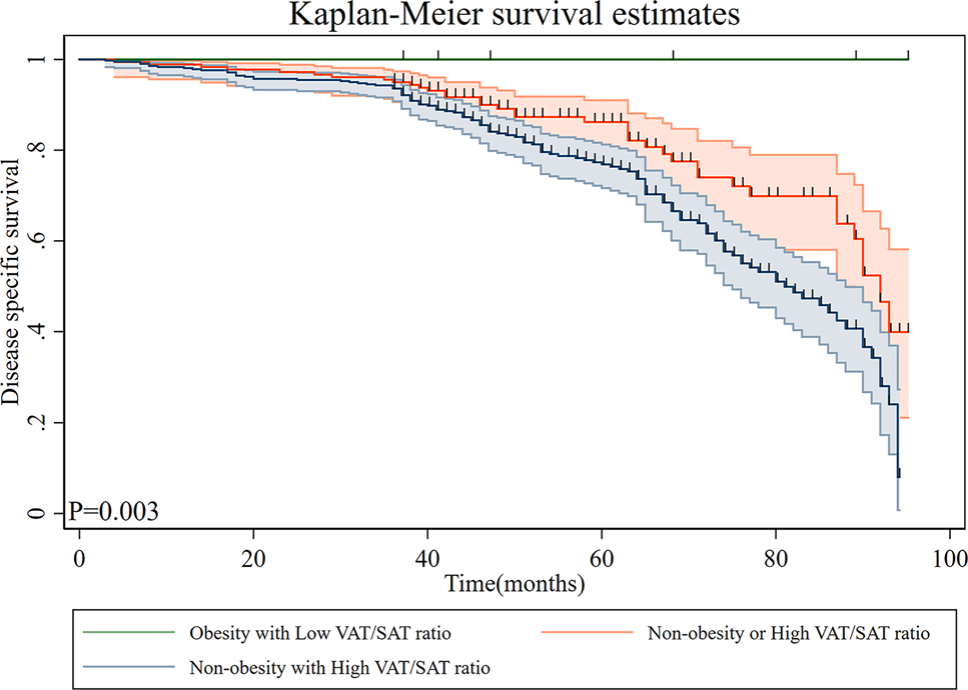
Disease-specific survival of patients with gastric cancer according to overweight/obesity and VAT/SAT ratio category. VAT, visceral adipose tissue; SAT, subcutaneous adipose tissue.

The patients were subclassified into three subgroups based on overweight/obesity and VAT/SAT categories. The characteristics of patients of each group are shown in **Table 4**. Compared with patients in the non-overweight/obesity with high VAT/SAT ratio group, most patients in the overweight/obesity with low VAT/SAT ratio were female and younger. Kaplan–Meier curves of DSS based on overweight/obesity and VAT/SAT categories are shown in **Figure 4**. There was a significant difference in DSS among the overweight/obesity and VAT/SAT categories groups (p = 0.003). Multiple Cox regression models were performed to explore the relationship between overweight/obesity and VAT/SAT categories and DSS (**Table 5**). Compared with patients in the other two groups, patients in the non-overweight/obesity with high VAT/SAT ratio group had a worse prognosis (HR = 1.89, 95% CI: 1.28–2.77).

**Table 4:** Characteristics of entire cohort and classified by overweight/obesity and VAT/SAT ratio.

	Overweight/obesity with low VAT/SAT ratio (N = 6)	Non-overweight/obesity or high VAT/SAT ratio (N = 180)	Non-overweight/obesity with high VAT/SAT ratio (N = 421)	P value
Age (>65 years)	1 (16.67)	65 (36.11)	202 (47.98)	**0.008**
Female	5 (83.33)	74 (41.11)	100 (23.75)	**<0.001**
Tumor location				0.283
Upper third	0 (0)	24 (13.33)	36 (8.55)	
Middle third	2 (33.33)	21 (11.67)	55 (13.06)	
Lower third	4 (66.67)	134 (74.44)	323 (76.72)	
Two-thirds or more	0 (0)	1 (0.56)	7 (1.66)	
Gastrectomy				0.863
Distal	5 (83.33)	142 (78.89)	341 (81)	
Total	1 (16.67)	38 (21.11)	79 (18.76)	
Proximal	0 (0)	0 (0)	1 (0.24)	
Tumor size (>5 cm)	1 (16.67)	43 (23.89)	104 (24.7)	0.970
Differentiated	4 (66.67)	101 (56.11)	208 (49.41)	0.252
Perineural invasion	3 (50.0)	69 (38.33)	167 (39.67)	0.791
Lymph vascular invasion	4 (66.67)	80 (44.44)	199 (47.27)	0.493
T category				0.304
T1	2 (33.33)	54 (30.0)	115 (27.32)	
T2	1 (16.67)	20 (11.11)	51 (12.11)	
T3	0 (0)	7 (3.89)	5 (1.19)	
T4a	3 (50.0)	93 (51.67)	243 (57.72)	
T4b	0 (0)	6 (3.33)	7 (1.66)	
N category				0.340
N0	4 (66.67)	78 (43.33)	173 (41.09)	
N1	0 (0)	26 (14.44)	68 (16.15)	
N2	2 (33.33)	27 (15.0)	91 (21.62)	
N3a	0 (0)	39 (21.67)	67 (15.91)	
N3b	0 (0)	10 (5.56)	22 (5.23)	
Chemotherapy	3 (50.0)	124 (68.89)	274 (65.08)	0.428

SAT, subcutaneous adipose tissue; VAT, visceral adipose tissue.

P value for difference among three groups in percentages (chi-square test or Fisher’s exact test) or rank sum (Kruskal–Wallis test).

Bold values represent statistically significant differences (P < 0.05).

**Table 5:** Association between non-overweight/obesity with high VAT/SAT ratio and disease-specific survival by multivariate Cox analysis in patients with gastric cancer.

	HR (95%CI)	P value
Non-overweight/obesity with high VAT/SAT ratio	1.89 (1.28, 2.77)	**0.001**
Age (>65 years)	1.57 (1.13, 2.17)	**0.007**
Female	0.95 (0.67, 1.34)	0.755
T category		
T1	1	
T2	2.44 (0.86, 6.94)	0.094
T3	2.88 (0.56, 14.93)	0.208
T4a	3.64 (1.48, 8.97)	**0.005**
T4b	10.78 (3.48, 33.36)	**<0.001**
N category		
N0	1	
N1	2.33 (1.18, 4.60)	**0.015**
N2	2.38 (1.27, 4.47)	**0.007**
N3a	6.62 (3.58, 12.24)	**<0.001**
N3b	8.93 (4.44, 17.94)	**<0.001**
Chemotherapy (Yes)	0.82 (0.54, 1.24)	0.351

SAT, subcutaneous adipose tissue; VAT, visceral adipose tissue; HR, hazard ratios; CI, confidence interval.

Bold values represent statistically significant differences (P < 0.05).

## Discussion

In the present study, we observed that in addition to the well-established T and N stages, BMI was an independent risk factor for the prognosis of resectable gastric cancer. The result showed overweight/obesity was a predictive factor for the prognosis of patients with gastric cancer. Subsequently, the adipose tissue distribution analysis showed that VAT was negatively correlated with the prognosis of gastric cancer, while SAT was protective correlated with the prognosis of gastric cancer. Moreover, the VAT/SAT ratio was an independent risk factor for the prognosis of gastric cancer. To the best of our knowledge, this is the first study documenting how different types of body adipose tissue distribution differentially influence the prognosis of patients with gastric cancer.

Meanwhile, our study showed that overweight/obese patients (BMI ≥25 kg/m^2^) had a better prognosis than non-overweight/obese patients (BMI <25 kg/m^2^) with gastric cancer. This phenomenon has been reported in some previous studies, although the sample size was small in those retrospective studies (25). However, a randomized controlled trial from Korea demonstrated that BMI was not associated with the prognosis of gastric cancer (26). Only 136 patients were included in the trial, including 27 obese cases, and the small sample size may be one of the reasons for the lack of statistically significant difference in the results. Meanwhile, Rodrigues reported that obesity was not associated with gastric cancer prognosis in the Western population, although the obesity threshold was BMI = 30 kg/m^2^ in the study (7). It is noteworthy that there are several differences in the study of gastric cancer between Eastern and Western countries. Firstly, compared with Western countries, Asian countries had a high incidence of gastric cancer, especially in China (27). Secondly, in Rodrigues’ study, obese gastric cancer patients in China accounted for less than 20% of the total number of cases, but over 60% accounted for Western obese gastric cancer patients, which may be the reason for the inconsistent results (7). Obese patients also have better physical and nutritional status than non-obese patients (28, 29). As a result, obese patients are more likely to receive adjuvant treatment, including chemotherapy, which may improve prognosis.

Although obesity (BMI ≥30 kg/m^2^) and overweight (BMI, 25–30 kg/m^2^) are not suitable for combined analysis, because the former is a real disease and the latter is only a risk factor. However, overweight and obesity were studied together in this study for two reasons. On the one hand, obesity accounts for a relatively small proportion of gastric cancer in Asian population. In this study, there were only seven obese patients. On the other hand, the target population was further divided into obese and overweight groups for subgroup analysis, and the trend obtained was consistent with the current results (**Table S1**). Therefore, obese and overweight patients were combined. In addition, skeletal muscle index (SMI) was analyzed, which is the ratio between skeletal muscle area (SMA) and height squared. In univariate analysis, HR = 1.024 (0.744, 1.409); p = 0.884, SMI was not associated with the prognosis of gastric cancer.

Obese patients may also have tumors that are sensitive to chemotherapy. Campbell et al. found that overweight and obese patients were more likely to have microsatellite instability-stable and low-microsatellite instability tumors than normal-weight patients in colorectal cancer (30). Evidence suggests that microsatellite instability-stable and low-microsatellite instability tumors are more susceptible to fluorouracil treatment than high-microsatellite instability tumors (31). This information may support the protective effect observed in overweight/obese patients. Therefore, the effect of obesity on cancer is complex and seemingly paradoxical. Most studies may use BMI to define overweight/obesity, which does not reflect adipose tissue distribution.

We further investigated the correlation between adipose tissue distribution and the prognosis of gastric cancer. The adipose tissue distribution was still associated with gastric cancer prognosis after adjusting for BMI. Consistent with our results, previous reports have shown the negative effect of visceral fat on the prognosis of cancer patients (11, 19). Similarly, Dong et al. included over 1,000 cases of gastric cancer and showed that low subcutaneous fat was a risk factor for the prognosis of gastric cancer, including overall survival and disease-free survival (32). However, some studies have suggested that visceral fat is not associated with the prognosis of gastrointestinal tumors. These studies had a sample size of less than 100 and most patients had received neoadjuvant chemotherapy (33–35). Neoadjuvant chemotherapy may affect the judgment of the influence of adipose tissue on the prognosis of gastric cancer. Besides, it is well known that TNM staging is the most important tool for prognostic stratification in gastric cancer. The TNM stage (ypTNM stage) of the patients after neoadjuvant therapy and the TNM stage (pTNM stage) of the patients who did not receive neoadjuvant therapy are not suitable to be combined for analysis, because the predictive value of the two for prognosis is inconsistent. Therefore, gastric cancer patients receiving neoadjuvant chemotherapy were not included in this study. Similarly, an observational study of 447 gastrointestinal tumors showed that pretreatment of subcutaneous adiposity was not associated with the prognosis of esophageal and gastric cancer (36). The study was limited in that it included only 65 cases of gastric cancer and did not perform a subgroup analysis of gastric cancer patients. A randomized controlled trial showed that preoperative visceral fat and subcutaneous fat areas were not associated with the prognosis of gastric cancer, although it had a small sample size (26). Conversely, Feng believed that low visceral fat was an independent risk factor for the prognosis of gastric cancer; however, only 46 cases of metastatic gastric cancer patients without surgery were included in the study (25). Therefore, these conclusions warrant further investigation.

VAT is an important metabolic tissue that secretes factors that systemically alter the immunologic, metabolic, and endocrine milieu. Excess VAT promotes chronic systemic inflammation with associated insulin resistance and dysmetabolism (37). Therefore, we further analyzed VAT and inflammatory markers, and the results showed that there was an association between the two. However, there was still a correlation between VAT and the prognosis of gastric cancer after adjusting inflammatory markers, suggesting that there could be other mechanisms by which VAT affects the prognosis of gastric cancer.

Furthermore, early studies suggested that patients with differentiated early gastric cancer had higher subcutaneous fat and visceral fat content than those with undifferentiated early gastric cancer and the researchers speculated that lower fat content was conducive to the occurrence and development of undifferentiated gastric cancer (38). Recently, visceral fat has been implicated in the promotion of carcinogenesis and cancer progression through several pathways, including adipocytokine-related inflammation and insulin resistance. The latter is associated with disturbances in insulin-like growth factor-1 (IGF-1) and hypoxia (39). Adipocytokines secreted by visceral adiposity attract inflammatory cells, particularly macrophages and T cells, which produce cytokines, such as the tumor necrosis factor-α and interleukin-6, thereby creating a proinflammatory, insulin-resistant, protumorigenic environment. Excess visceral fat decreases adiponectin. Adiponectin inhibits the proliferation, angiogenesis, and inflammatory properties of tumor cells and promotes their apoptosis (40). It also induces chronic hyperinsulinemia followed by insulin resistance, which reduces the expression of IGF-binding protein, subsequently increasing IGF-1 expression. IGF-1 has protumorigenic properties and is linked to increased malignancy and progression of several gastrointestinal malignancies (41).

Other studies reported that the VAT/SAT ratio is associated with surgical site infections in patients with gastric cancer (42). Subcutaneous fat is associated with a postoperative incisional hernia for gastric cancer (43). Higher visceral fat was associated with higher postoperative complications of gastric cancer (44). However, there is growing evidence that postoperative complications are associated with the long-term prognosis of gastric cancer. A possible explanation is that cell-mediated immunity is compromised by surgical stress and excessive catecholamine and prostaglandin responses adversely affect the immune system, contributing to metastatic progression and worse survival outcomes (45).

Finally, we grouped patients with resectable gastric cancer using the VAT/SAT ratio and BMI as stratification factors. The results showed that overweight/obese patients with a low VAT/SAT ratio had the best prognosis, although only six cases were involved. In contrast, non-overweight/obese patients with a high VAT/SAT ratio had the worst prognosis. The latter accounted for the highest percentage of the entire cohort. Therefore, we believe that besides BMI, the adipose tissue distribution should also be considered during preoperative evaluation of gastric cancer patients.

This study comprehensively elaborated on the influence of different adipose tissue distributions on the prognosis of gastric cancer. We believe that BMI is a prognostic factor, and VAT and SAT have different effects on the prognosis of gastric cancer. However, this study has some shortcomings. First, we did not subdivide overweight/obese patients into subgroups according to severity of obesity, such as super-obese patients. However, super obesity is rare in patients with gastric cancer, especially in Asian populations. Secondly, because data on changes in adipose tissue distribution after surgery are not available, we did not analyze the changes in adipose tissue distribution after surgery and their association with prognosis, which merit further exploration. Finally, due to the initial stage of relevant research, the threshold value was not uniformly standardized. However, our method to establish an optimal cutoff value is adopted by most of the current studies. Further research is warranted to verify our conclusions.

## Conclusions

Prognostic factors for resectable gastric cancer include overweight/obesity. Further, VAT and SAT have different effects on the prognosis of gastric cancer. Our results showed that overweight/obesity is a protective factor for the prognosis of gastric cancer. The VAT/SAT ratio could be a promising prognostic factor for gastric cancer. Therefore, in preoperative evaluation of gastric cancer patients, attention should be paid not only to BMI but also to adipose tissue distribution.

## Data Availability Statement

The raw data supporting the conclusions of this article will be made available by the authors, without undue reservation.

## Ethics Statement

Written informed consent was obtained from the individual(s) for the publication of any potentially identifiable images or data included in this article.

## Author Contributions

LG and YZ conceived and contributed to design of the study. JH, BX and LY collected and analyzed the data. KY and JZha analyzed and interpreted the data. PC, JZhe and JL supervised and contributed to writing the manuscript. All authors contributed to the article and approved the submitted version.

## Funding

This study was funded by the Key Laboratory of Diagnosis and Treatment of Digestive System Tumors of Zhejiang Province (Grant No. 2019E10020), Ningbo Clinical Research Center for Digestive System Tumors (Grant No. 2019A21003) and Ningbo Municipal Non-Profit Fund for Applied Research (Grant No. 2019F1033).

## Conflict of Interest

The authors declare that the research was conducted in the absence of any commercial or financial relationships that could be construed as a potential conflict of interest.

## Supplementary Material

Supplementary Figure 1 Optimal cutoff values of adipose tissue distribution parameters. **(A)** SAT percentage; **(B)** VAT percentage; **(C)** VAT/SAT ratio. VAT, visceral adipose tissue; SAT, subcutaneous adipose tissue.

## Abbreviations

BMI, body mass index; VAT, visceral adipose tissue; SAT, subcutaneous adipose tissue; DSS, disease-specific survival; CT, computed tomography; SMI, skeletal muscle index; SMA, skeletal muscle area; IGF-1, insulin-like growth factor-1.
