# Supplementary figures and images for: Prognostic Value of Pretreatment Overweight/Obesity and Adipose Tissue Distribution in Resectable Gastric Cancer: A Retrospective Cohort Study
Source: Front Oncol. 2021 Jun 24;11:680190. doi: 10.3389/fonc.2021.680190 (PMC8264507; doi:10.3389/fonc.2021.680190)

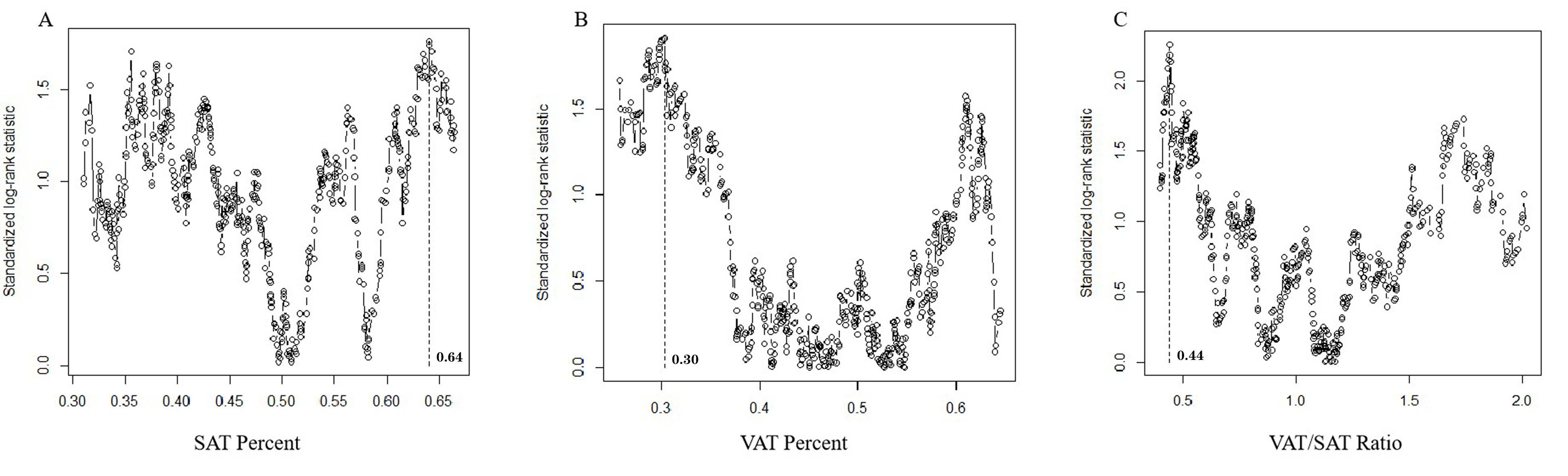

Supplement: Supplementary Figure 1 — Optimal cutoff values of adipose tissue distribution parameters. (A) SAT percentage; (B) VAT percentage; (C) VAT/SAT ratio. VAT, visceral adipose tissue; SAT, subcutaneous adipose tissue. [file Image_1.tif]
