# Supplementary material for: Prognostic Value of Pretreatment Overweight/Obesity and Adipose Tissue Distribution in Resectable Gastric Cancer: A Retrospective Cohort Study
Source: Front Oncol. 2021 Jun 24;11:680190. doi: 10.3389/fonc.2021.680190 (PMC8264507; doi:10.3389/fonc.2021.680190)
Supplement: Supplementary file 2 [file Table_1.docx]

**Table S1. Association between BMI category and disease-specific survival by multivariate Cox analysis in patients with gastric cancer.**

|  | HR (95%CI) | P value |
| --- | --- | --- |
| **BMI category** |  |  |
| BMI<25 | 1.00 |  |
| 25≤BMI<30 | 0.70 (0.43, 1.15) | 0.158 |
| BMI≥30 | 0.16 (0.02, 1.19) | 0.073 |
| Age (>65 years) | **1.57 (1.13, 2.17)** | **0.007** |
| Female | 0.92 (0.65, 1.30) | 0.619 |
| T category |  |  |
| T1 | 1.00 |  |
| T2 | 2.47 (0.87, 7.03) | 0.091 |
| T3 | 2.67 (0.51, 14.07) | 0.246 |
| T4a | **3.82 (1.55, 9.40)** | **0.004** |
| T4b | **10.54 (3.41, 32.61)** | **<0.001** |
| N category |  |  |
| N0 | 1.00 |  |
| N1 | **2.39 (1.21, 4.72)** | **0.012** |
| N2 | **2.31 (1.23, 4.34)** | **0.009** |
| N3a | **6.15 (3.33, 11.34)** | **<0.001** |
| N3b | **8.85 (4.41, 17.76)** | **<0.001** |
| Chemotherapy | 0.81 (0.54, 1.23) | 0.321 |

BMI, Body mass index; HR, Hazard ratios; CI, Confidence interval.
